# Self-referred walk-in patients in the emergency department – who and why? Consultation determinants in a multicenter study of respiratory patients in Berlin, Germany

**DOI:** 10.1186/s12913-020-05689-2

**Published:** 2020-09-10

**Authors:** Felix Holzinger, Sarah Oslislo, Martin Möckel, Liane Schenk, Mareen Pigorsch, Christoph Heintze

**Affiliations:** 1 Charité – Universitätsmedizin Berlin, corporate member of Freie Universität Berlin, Humboldt-Universität zu Berlin, and Berlin Institute of Health, Institute of General Practice, Charitéplatz 1, 10117 Berlin, Germany; 2 Charité – Universitätsmedizin Berlin, corporate member of Freie Universität Berlin, Humboldt-Universität zu Berlin, and Berlin Institute of Health, Division of Emergency Medicine, Berlin, Germany; 3 The College of Public Health, Medical and Veterinary Sciences, James Cook University, Townsville, QLD Australia; 4 Charité – Universitätsmedizin Berlin, corporate member of Freie Universität Berlin, Humboldt-Universität zu Berlin, and Berlin Institute of Health, Institute of Medical Sociology and Rehabilitation Science, Berlin, Germany; 5 Charité – Universitätsmedizin Berlin, corporate member of Freie Universität Berlin, Humboldt-Universität zu Berlin, and Berlin Institute of Health, Institute of Biometry and Clinical Epidemiology, Berlin, Germany

**Keywords:** Emergency department, Respiratory conditions, Consultation determinants, Health care utilization

## Abstract

**Background:**

Emergency department (ED) consultations are on the rise, and frequently consultations by non-urgent patients have been held accountable. Self-referred walk-in (SRW) consulters supposedly represent a predominantly less urgent patient population. The EMACROSS study aimed to explore consultation determinants and motives in SRW patients with respiratory symptoms.

**Methods:**

Multicenter survey of adult ED patients with respiratory complaints in eight emergency departments in central Berlin, Germany. Secondary hospital records data including diagnoses was additionally assessed. Characteristics of SRW and non-SRW patients were compared. Determinants of SRW consultation were evaluated by binary logistic regression. Consultation motives were analyzed descriptively. As a supplemental approach, network analysis (lasso-regularized mixed graphical model) was performed to explore connections between consultation determinants, consultation features and motives.

**Results:**

Between June 2017 and November 2018, *n* = 472 participants were included, the median age was 55 years (range 18–96), 53.2% of patients were male and *n* = 185 cases (39.2%) were SRW consulters. The SRW group showed lower proportions of potentially severe (pneumonia and respiratory failure, *p* < 0.001, χ^2^ test) and chronic pulmonary conditions. Determinants of SRW consultation identified by logistic regression were younger age (*p* < 0.001), tertiary education (*p* = 0.032), being a first-generation migrant (*p* = 0.002) or tourist (*p* = 0.008), having no regular primary care provider (*p* = 0.036) and no chronic pulmonary illness (*p* = 0.017). The area under the curve (AUC) for the model was 0.79. Personal distress and access problems in ambulatory care were stated most frequently as consultation motives in the SRW group; network analysis showed the scarcity of associations between demographic and medical SRW determinants and motives triggering the actual decision to consult.

**Conclusions:**

As to “who” consults, this study identified demographic and medical predictors of SRW utilization. The said markers seem only remotely connected to “why” people decide for SRW visits. To alleviate ED crowding by addressing frequent SRW consultation motives, interventions focused on the ability for symptom self-assessment and at better-accessible alternative care seem sensible.

**Trial registration:**

German Clinical Trials Register (DRKS00011930); date: 2017/04/25.

## Background

Emergency department (ED) consultations are rising in many countries [1, 2]. A considerable proportion is managed on an outpatient basis [3]. ED utilization for non-urgent complaints – which could alternatively be adequately managed by a general practitioner (GP) – has become a much-discussed issue in the context of ED crowding [4, 5]. ED overburdening is supposed to contribute to a lack of care resources for actually critical patients, ultimately adding to adverse outcomes and even increased mortality [2]. In the discussion of non-urgent ED utilization, a notion frequently expressed especially by health professionals is an alleged misuse of emergency care structures by irresponsible consumers, but perceptions of patients may differ considerably [6]. Understanding care demands as well as consultation patterns and triggers is thus of vital importance to allow for developing sensible solutions to the pressing problem of ED overuse: we need to better comprehend who these consulters are, what groups of society they belong to, and what they hope to gain by turning to the ED with certain complaints. This may greatly help in devising future care structures both demand-oriented – as acceptance on the patient side is central – and resource-sparing.

Utilization motives of non-urgent ED patients have been evaluated in various settings [7, 8]. Alleged contributing factors include perceived severity of symptoms, health-related anxiety, as well as considerations of convenience [3, 7, 9]. This is considered to be linked with organizational access barriers in primary care (PC) [6, 10]. Many studies have included heterogeneous populations [7] with diverse consultation triggers, ranging from minor injuries to gastrointestinal or cardiorespiratory complaints [3, 11]. However, ED visits and how they come about may vary considerably depending on the nature of symptoms [12]. Medical issues like thoracic pain or subjective dyspnea for example, although not always caused by serious disease, may be associated with greater worry, uncertainty – and thus subjective urgency – than e.g. acute musculoskeletal ailments or skin problems [3], simply due to the not straightforward constellation. Such less clear-cut situations, in which patients need to self-assess symptoms and then decide whether to visit an ED, constitute the most interesting cases when wanting to understand what drives utilization patterns. For this purpose, respiratory complaints constitute an ideal model, as they are very frequent consultation reasons in EDs as well as in PC [3, 13, 14] and their underlying reasons encompass a wide spectrum, ranging from more serious (e.g. pneumonia) to non-serious (e.g. common cold) as well as acute and chronic conditions [15, 16].

Concerning purportedly less urgent ED visits, means of arrival provide a first indicator: walk-in patients are presumably less severely ill than those arriving by ambulance [17], as in the latter the necessity of ED treatment will usually have been either determined by a health care professional (e.g. referring physician), or the patient will have felt too severely afflicted to consider other transportation. Among walk-in consulters, patients who decided to visit the ED on their own accord as self-referrals constitute the most interesting population for studying ED consultation reasons and associated factors [12, 18].

To gain a deeper understanding of ED utilization determinants in a population with an exemplary symptomatology, we aimed to comprehensively explore demographic and medical characteristics as well as consultation motives of self-referred walk-in ED patients presenting with respiratory symptoms.

## Methods

### Overview: research network and study

The multicenter mixed methods EMACROSS (Emergency and Acute Care for Respiratory Diseases beyond Sectoral Separation) study investigates characteristics, motives and health care utilization of patients with respiratory symptoms in a network of eight EDs in the central district of Berlin, Germany (Berlin-Mitte). It is a subproject of EMANet (Emergency and Acute Medicine Network for Health Care Research), which focuses on acute care for a number of model conditions selected in the context of the Ambulatory Care Sensitive Conditions (ACSC) concept [19].

EMACROSS consists of a quantitative two-stage survey of respiratory ED patients, an evaluation of secondary hospital data, and a qualitative module [20]. This paper reports the results of the t0 survey and analysis of hospital records. The protocol was registered a priori in the German Clinical Trials Register (DRKS00011930). The study was approved by the ethics committee of Charité – Universitätsmedizin Berlin (EA1/361/16).

### Setting and timeframe

Participants were recruited in our network comprising the entirety of EDs in the district, including two university medical centers, between 1st of June 2017 and 30th of November 2018. Patients were assessed for eligibility at presentation based on symptoms reported to the triage officer. If inclusion criteria were met, written informed consent was obtained. Recruitment was conducted regularly from Monday to Friday between 9 am and 5 pm and intermittently in the evenings and on weekends. The focus of recruitment was placed on regular physicians’ office hours due to our interest in choosing the ED versus conceivable alternative care, ED self-referral being not at all limited to out-of-hours periods [12]. The survey was conducted during waiting times or between investigations.

### Inclusion and exclusion criteria

Patients of both sexes aged ≥18 years with respiratory symptoms (e.g. cough, dyspnea etc.) were included. An initially envisaged diagnosis-based enrollment [21] was abandoned as unfeasible after pilot testing due to the characteristics of ED care, definite diagnoses being available only late in the visits and outpatients frequently desiring to leave immediately after receiving their discharge letter. Patients were excluded if unable (e.g. as to dementia or severity of acute condition) or unwilling to consent, or lacking adequate proficiency in one of the questionnaire languages (German, English, Turkish, and Arabic). Recruitment was initially limited to outpatients. This proved problematic in the study workflow: patients had to be interviewed at a time when it was frequently undecided whether they would be ultimately admitted. In order to avoid having to exclude patients after completed interviews and thus losing valuable data, recruitment was extended to eventual inpatients as of October 2017; the study protocol was thus amended.

### Data collection

The questionnaire assessed demographic and medical characteristics as well as consultation motives and health care utilization [21]. Items were derived from established instruments where available and appropriate. Assessment of health care utilization was based on the German Health Interview and Examination Survey for Adults (DEGS) [22], the PHQ4 questions were included as indicators of mental health [23], general life satisfaction was measured with the short scale L-1 [24], and education was assessed corresponding to the CASMIN classification [25]. Other items were specifically developed for EMANet. The final survey contained 43 questions (plus eventual sub-items). The German and English language versions of the questionnaire are available as Additional file 1 and Additional file 2. Several pre-test rounds were carried out [26].

The questionnaire was tablet-based, data was entered by study nurses conducting face-to-face interviews. A few questions, e.g. for assessment of ED consultation motives, were posed openly and study personnel matched answers to a list of pre-formulated options. Free text documentation was used in cases of no match to the list. Concerning consultation motives, patients could thus freely relate their considerations; a combination of several reasons could potentially apply (multi-response data). Personnel received precise instructions, interpretation aids and repeated interviewer trainings. Data was directly transferred to a secure database server.

Additionally, medical (e.g. triage, symptoms, diagnoses) and administrative (e.g. admission, discharge) data was extracted from hospital records via electronic case report form (eCRF). For quality assurance, random double entries of 5% of cases were performed and collated.

Three months after the baseline survey, a telephone or postal follow-up ensued to longitudinally assess health and utilization [21]. Follow-up data is currently analyzed.

### Data analysis

#### Definitions, variables and data preparation

##### Target group: self-referred walk-in (SRW) patients

We delimited self-referred patients as cases in which no medical professional or institution was involved in the visit’s initiation, namely participants not referred by a physician, hospital or department, or nursing home staff. Walk-in cases were defined as patients reporting arrival by any means (e.g. by foot, car, public transport) other than emergency medical services (EMS) or ambulance transport, and hospital records neither indicating such. SRW patients were defined as cases with both characteristics, as compared to non-SRW.

##### Patient characteristics

Data on medical and demographic characteristics was primarily derived from the t0 survey. Most variables directly correspond to the respective survey questions. For some ordinal variables, categories were combined, e.g. in case of small subgroups or if otherwise deemed theoretically reasonable, e.g. a variable on previous frequency of similar symptoms, which was collapsed into “new symptoms” vs. “prior existence of comparable symptoms”. Ordinal variables with a substantial number of classes (e.g. 0–10 scales) were interpreted as continuous [27]. A summary variable for symptom-associated distress was created by combining scales assessing components of this construct (severity and associated threat) [28, 29] by calculating the average of the two scale values. The eight-level CASMIN education scale was collapsed into three levels of low, intermediate, and high educational attainment [30]. The “low” level comprised CASMIN levels 1a to 1c (primary + low secondary), the “intermediate” level 2a to 2c (intermediate + high secondary) and the “high” level 3a and 3b (tertiary education). For chronic pulmonary morbidity, hospital records and survey data were combined to enhance validity [31]. When cross-tabulating the dichotomous variables for both data sources (chronic pulmonary condition mentioned: yes/no), concordance was moderate at a Cohen’s kappa of 0.5 [32], which is comparable to the literature [33, 34]. We considered a chronic pulmonary condition as likely present if this was either self-reported or a corresponding diagnosis documented. Patients with two or more chronic conditions were defined as multimorbid [35].

##### Consultation motives

Consultation motives were grouped into thematic summary categories based on pertinent classes of the framework of Coster et al. [36]. Categories were labeled as “distress”, “access”, “quality” and “convenience” (Table 1). “Distress” encompasses all answers relating to symptom severity and anxiety, “access” covers issues of service–defined barriers to alternative care (e.g. appointment availability and office hours in PC), as well as situations of patients not knowing who to contact (e.g. visitors ignorant of local health care). “Quality” summarizes expectations of better care in the hospital setting, and the “convenience” theme comprises patient-defined considerations regarding comfort and ease of ED access.

**Table 1:** Consultation motive groups and examples of source items

Motive group	Number of source items	Examples
Distress	2	“Because the situation felt threatening to me” “Because my complaints were so severe”
Access	5	“Because my GP’s practice was closed” “Because I could not get a timely appointment with my GP or specialist, although I tried to.” “Because I am just visiting this city”
Quality	7	“Because diagnostic and therapeutic options are more comprehensive in the hospital” “Because there are special experts in the hospital” “Because the results of investigations are available more quickly”
Convenience	5	“Because the ED is always open and no appointment is necessary” “Because the ED is closer to my home than a practice”

*Note*. Question posed to the participants was “Why did you decide to visit an emergency department with your current complaints?”

##### ED consultation features and outcomes

Information on time of presentation, hospital admission, triage and diagnoses was available from hospital records. Triage categories of the Manchester Triage System were combined in a binary variable delineating “high urgency” (levels 1, 2, and 3) and “low urgency” (levels 4 and 5) analogous to van der Linden et al. [12]. Concerning presentation time, we distinguished office-hours from out-of-hours based on usual opening times of GPs’ practices. “Out-of-hours” was defined as 6.00 pm to 8.00 am on weekdays, plus all weekends. As German practices usually close on Wednesday afternoons, an extended out-of-hours timeframe starting at 2.00 pm was defined for this day.

#### Statistics

Patient characteristics as well as consultation motives were summarized descriptively. For logistic regression of SRW determinants, a set of potential predictors and control variables was compiled, based on theoretical plausibility and the literature. The number of candidate predictors was limited by events per variable (EPV) to avoid bias, a current recommendation being to aim for EPV of 15 or higher [37]. We carried out univariate statistics and noted which variables showed significance at a relaxed level (*p* ≤ 0.25) [38]. However, non-significance did not result in immediately discarding a predictor. For non-significant variables, we carefully considered their potential importance for the model as e.g. control variables, in which case they were retained. This could also be the case if the variable had been identified as an important predictor in a previous study. A first multivariate model was constructed. We then checked the effects of discarding single predictors on the variable set and assessed fit and predictive accuracy of the candidate models to decide which variables to include in the final set. Model fit was assessed by the Hosmer-Lemeshow test; Cook’s distance was used to investigate for influential outliers. Classification was assessed by the area under the receiver operating characteristic (ROC) curve. Effect sizes are reported as odds ratios with 95% confidence intervals.

We did not conduct an automated variable selection procedure like stepwise regression, as to avoid the risk of obtaining a biased model with falsely narrow confidence intervals and low *p* values [39, 40]. To overcome the problems of stepwise methods, several solutions to variable selection have been proposed, markedly focusing on expert knowledge and theory rather than strict significance thresholds, which was the approach chosen for our logistic regression analysis. Alternatively, variable selection by newer statistical techniques encompassing penalization and shrinkage – like ridge or LASSO (least absolute shrinkage and selection operator) regression – would be preferable to conventional automated selection methods in many constellations [37]. A LASSO approach was part of our network analysis, which is outlined further down in this methods section.

For group comparisons of categorical variables, the χ^2^ test was used. The significance level for all analyses was set at 0.05. Descriptive statistics and regression were performed in IBM SPSS Version 25 and R (JASP 0.11.1 interface) [41].

For explorative investigation and visualization of patient characteristics in connection with consultation motives as well as ED consultation features and outcomes, a network analysis of complete case data was conducted. In such networks, variables are labeled as “nodes” and connections as “edges”. Analyses were performed with the R packages “mgm” [42] and “bootnet” [43]. The “mgm” package allows estimation of k-degree mixed graphical models (MGM) via regularized neighborhood regression; this method was suitable as the analysis included numerical as well as categorical variables. The MGM was estimated using LASSO regularization, which sets very small parameter estimates to exactly zero and returns sparse – and thus conservative – network models [43]. The LASSO utilizes a tuning parameter to control the degree of regularization, which was selected by minimization of the Extended Bayesian Information Criterion (EBIC) [44]. EBIC penalizes solutions that involve more variables and more neighbors of nodes, with a hyperparameter γ determining the strength of the extra penalty on the number of neighbors [45]. This hyperparameter was set to 0.25 (default in “mgm”) [42]. Either of the estimates was required to be nonzero for an edge to be present (OR-rule) [45, 46]. The network was plotted via the R package “qgraph” [47]. Node placement is determined by the Fruchterman-Reingold algorithm which places nodes such that all the edges are of more or less equal length while aiming to avoid edges crossing. Edge width is proportional to the edge-weight, green edges indicate positive relationships and red edges negative relationships [42]. To avoid edges without visual indication of a sign, multi-categorical variables (education, migration and travel) were binarized by combining categories as suggested by the preceding regression analysis. We assessed predictability of SRW by connected nodes, defined as correct classification beyond the marginal [48]. Accuracy of edge-weights was evaluated by nonparametric bootstrapping via “bootnet” [43].

## Results

### Study cohort

A total of *n* = 472 cases were included, while *n* = 1121 initially screened patients had to be excluded. Exclusion reasons and frequencies are shown in Table 2. Details on recruitment monitoring and non-responder analysis in EMANet have also been published elsewhere [49].

**Table 2:** Potential participants: screening, exclusion frequencies and reasons

Patients	n	For exclusions: % of n = 1121 patients excluded
**Screened**	1593	–
**Included**	472	–
**Excluded**	1121	100.0
Unable to give valid informed consent (e.g. cognitive impairment)	180	16.1
Case definition criteria not met (e.g. wrong symptom, age etc.)	187	16.7
Acute medical reasons	42	3.7
Inadequate language proficiency	151	13.5
Restrictions of ED workflow (e.g. unavailable as to ongoing treatment)	262	23.4
Refusal	299	26.7
- Feeling too ill	176	
- General disinterest	98	
- Other reasons for refusal	25	

Required data to determine SRW vs. non-SRW status was available for *n* = 463, of which 185 (40.0%) were classified as SRW. For nine cases, necessary information for classification was missing. The frequencies of all combinations of the variables defining the target group are reported in Table 3, while Table 4 shows characteristics of the total cohort and SRW vs. non-SRW cases.

**Table 3:** Defining variables of target group SRW: initiation of visit and means of arrival at the ED

Variable	Self-referred	Referred by health professional	Referral unknown	Total
Walk-in	185^a^	103^b^	3	291
EMS/ambulance	102^b^	66^b^	3^b^	171
Means of arrival unknown	5	4^b^	1	10
Total	292	173	7	472

*Note.* Numbers in table represent cases in groups; EMS/ambulance patients: defined as referred if emergency service/transport initiated by a health professional, and self-referred if initiated by the patient; The SRW target group is marked with ^a^, constellations classified into the non-SRW group with ^b^

**Table 4:** Characteristics of study participants

		Group		
Variable	Measure	Total cohort	SRW	non-SRW
**Participants**	n	472	185	278
***Demographics***				
**Age**	n	472	185	278
	Mean (SD) Median (Range)	53.6 (19.2) 55.0 (18–96)	44.9 (17.2) 42.0 (18–96)	59.7 (18.0) 62.5 (19–92)
**Sex**	n	472	185	278
Male	%	53.2	47.0	57.6
Female	%	46.8	53.0	42.4
**Migration and travel**	n	466	185	273
Migrant first generation	%	21.9	35.1	12.8
Second generation	%	6.9	8.1	5.9
Tourist	%	4.3	8.6	1.5
**Education (CASMIN)**	n	463	183	273
Low	%	25.5	15.8	32.2
Intermediate	%	43.6	39.9	45.1
High	%	30.9	44.3	22.7
***ED consultation***				
**Means of arrival**	n	462	185	274
Walk-in	%	63.0	100.0	37.6
EMS	%	30.7	0.0	51.8
Ambulance transport	%	6.3	0.0	10.6
**Initiation of visit**	n	465	185	275
Self-referred	%	62.8	100.0	37.1
Health professional	%	37.2	0.0	62.9
**Triage category**	n	456	180	267
Lower urgency	%	41.9	53.9	33.7
Higher urgency	%	58.1	46.1	66.3
**Time of presentation**	n	472	185	278
Out-of-hours visit	%	17.2	18.4	15.8
During office hours	%	82.8	81.6	84.2
***ED symptoms***				
**Symptom novelty**	n	467	184	275
New symptoms	%	36.4	39.7	34.9
Recurrent symptoms	%	63.6	60.3	65.1
**Symptom-associated distress**	n	442	177	259
	Mean (SD) Median (Range)	7.2 (1.8) 7.5 (1.5–10)	7.0 (1.8) 7.0 (1.5–10)	7.3 (1.8) 7.5 (2–10)
***Chronic conditions and care***				
**Chronic pulmonary condition**				
Self-reported	n	465	183	275
	yes: %	48.6	31.7	60.0
Hospital record	n	472	185	278
	yes: %	43.2	34.1	48.9
Combined	n	467	184	276
	yes: %	58.7	44.0	68.1
**Multimorbidity**	n	465	183	275
	yes: %	53.5	39.9	63.3
**Attached to GP**	n	464	183	275
	yes: %	86.6	76.5	93.5
**ED visit in past 6 months**	n	453	179	268
	yes: %	34.2	28.5	37.7
***Mental health***				
**PHQ4 anxiety subscale**	n	467	185	275
	Mean (SD) Median (Range)	1.7 (1.9) 1.0 (0–6)	1.9 (1.9) 1.0 (0–6)	1.6 (1.8) 1.0 (0–6)
**PHQ4 depression subscale**	n	467	185	275
	Mean (SD) Median (Range)	2.2 (2.2) 2.0 (0–6)	2.2 (2.2) 2.0 (0–6)	2.2 (2.2) 2.0 (0–6)
**General life satisfaction**	n	457	183	268
	Mean (SD) Median (Range)	6.9 (2.6) 8.0 (0–10)	6.9 (2.5) 8.0 (0–10)	7.0 (2.7) 8.0 (0–10)

*Note*. n = cases with available data for respective characteristic; % = percentage of cases with available data; Migration and travel: first generation = not born in Germany, second generation = participant born in Germany and mother/father (or both) born in another country; General life satisfaction, subjective symptom-associated distress: 0–10 scales; PHQ4 anxiety and depression: 0–6 subscales; Chronic pulmonary morbidity combined: if either self-reported or documented in hospital records

The SRW group was younger and included a greater proportion of females, migrants as well as tourists. SRW patients also showed higher formal education status and less urgent triage, whereas the proportion of out-of-hours consultations was similar in both groups. Morbidity in general and concerning chronic pulmonary conditions was higher in non-SRW patients, while groups did not differ markedly regarding mental health.

### Predictors of SRW consultations

Potential determinants of SRW consultations were evaluated by logistic regression. Results of the multivariate model are shown in Table 5. The Hosmer-Lemeshow test did not show significance (*p* = 0.969, χ^2^ = 2.337, df = 8), thus supporting model fit.

**Table 5:** Logistic regression model for SRW vs. non-SRW as dependent variable (*n* = 438 complete cases)

Independent variable	Coefficient B	Standard error	p value	Odds ratio	95% CI lower bound	95% CI upper bound
**Age**	−0.030	0.007	0.000	0.970	0.957	0.984
**Sex** *Reference: female*	−0.418	0.233	0.073	0.659	0.417	1.039
**Migration and travel** *Reference: no related feature*			0.002			
Migrant first generation	0.848	0.279	0.002	2.336	1.351	4.040
Second generation	0.030	0.441	0.946	1.030	0.434	2.445
Tourist	1.669	0.634	0.008	5.309	1.533	18.390
**Education (CASMIN)** *Reference: low*			0.041			
Intermediate	0.126	0.312	0.686	1.134	0.616	2.090
High	0.700	0.326	0.032	2.014	1.063	3.819
**Triage category** *Reference: lower urgency*	−0.227	0.237	0.337	0.797	0.501	1.268
**Out-of-hours visit**	0.205	0.303	0.497	1.228	0.679	2.222
**Chronic pulmonary condition**	−0.558	0.234	0.017	0.572	0.362	0.906
**Attached to GP**	−0.736	0.352	0.036	0.479	0.240	0.955

*Note*. Combined variable for chronic pulmonary condition

Being a first-generation migrant or a tourist and having a high level of education (tertiary) were identified as predictive of SRW in the multivariate analysis. Higher age, having a chronic pulmonary condition and being regularly attached to a GP practice lowered the probability of an SRW consultation. Sex and out-of-hours were retained in the model as control variables. Triage category was not shown as independently predictive in the multivariate model; the same applies to other variables evaluated during model building (previous utilization, multimorbidity, symptom-associated distress). ROC curve analysis showed an AUC (area under the curve) of 0.79 for the model.

### Consultation outcomes

Compared to non-SRW, a considerably greater proportion of SRW cases were managed as ED outpatients. Concerning ED and hospital diagnoses, the non-SRW group showed higher proportions of pneumonia and COPD than SRW patients did. Respiratory failure was documented in 27.7% of non-SRW cases, compared to only 7.0% in SRW patients. In non-SRW patients admitted to hospital, the proportion of respiratory failure diagnosed was also higher than in SRW patients admitted (52.4%, vs. 34.2%, *p* = 0.046). SRW patients had a higher share of upper airway condition diagnoses coded, as well as asthma. Consultation outcomes are summarized in Table 6.

**Table 6:** Outcomes of ED consultation

		Group			
Variable	Measure	Total cohort	SRW	non-SRW	Group difference
**Participants**	n	472	185	278	χ2 test: p value
**Respiratory diagnoses, ICD-10 codes**					
Pneumonia J12-J18	%	23.3	12.4	30.6	< 0.001
COPD and chronic bronchitis J40-J44	%	34.3	20.5	43.2	< 0.001
Asthma bronchiale J45-J46	%	9.7	14.1	6.8	0.010
Other respiratory tract infection (incl. bronchitis, influenza) J09-J11, J20-J22	%	8.5	10.8	7.2	0.175
Upper airway conditions J0x/J3x	%	10.2	16.2	6.5	0.001
Respiratory symptom diagnosis only (ICD-10 R section code, no J section diagnosis)	%	14.4	17.8	11.9	0.072
Respiratory failure J96 coded	%	19.5	7.0	27.7	< 0.001
**ED visit consequence**					
Outpatients	%	61.2	79.5	48.6	< 0.001
Hospital admission	%	38.8	20.5	51.4	

*Note*. Data on visit outcomes available for all participants; % = percentage of cases; Diagnoses: Respiratory ICD-codes documented in ED and/or hospital documentation: ED documentation only for outpatients, discharge diagnoses additionally considered for inpatients. Multiple diagnoses possible for individual cases

### Consultation motives

In the framework of motive groups, “distress” was the main consultation reason voiced by both SRW and non-SRW patients, although by a greater proportion of the SRW group. “Access” constituted the second most important motive group, with a considerably greater share of SRW patients relating such. The same applies, to a somewhat lesser extent, to “quality” and “convenience” (Table 7).

**Table 7:** Motive groups in SRW/non-SRW patients

	SRW, % of *n* = 185	non-SRW, % of *n* = 278	Overlap of categories (SRW group only, percentage of total positives in row)			
Motive group			Distress	Access	Quality	Convenience
Distress	65.9	50.0		37 (30.3%)	22 (18.0%)	3 (2.5%)
Access	38.4	10.4	37 (52.1%)		14 (19.7%)	4 (5.6%)
Quality	24.3	9.7	22 (48.9%)	14 (31.1%)		4 (8.9%)
Convenience	5.4	2.2	3 (30.0%)	4 (40.0%)	4 (40.0%)	

*Note.* Multiple responses allowed; motives in one or more of these groups were reported by *n* = 174 (94.1%) of the SRW group and by *n* = 163 (58.6%) of the non-SRW group

Inherent to their assessment as multi-response data, categories partially overlap, which is outlined for the SRW group in Table 7. For example, about half of patients in the “access” and “quality” groups also related “distress” as additional consultation motive.

As to better understand possible access problems in ambulatory care, a more detailed evaluation of the reasons reported by SRW patients was conducted. Unavailability of practices (out-of-hours, weekend, vacation) as well as difficulties in getting timely appointments were most prominently reported (35.2% and 32.4% of *n* = 71 participants in the “access” motive group). Of the 25 cases stating that they could not reach their physician, eleven (44.0%) presented to the ED out-of-hours, 14 (56.0%) during regular office hours. Patients were additionally asked whether they had tried to contact a doctor’s practice prior to visiting the ED; the proportion reporting such was higher in the non-SRW group than in SRW patients (59.9% vs. 41.1%, *p* < 0.001). For SRW patients, the access problems related suggest a substantial share of unsuccessful contact attempts. On the other hand, non-SRW patients’ better accomplishment in this regard will supposedly frequently have resulted in their eventually being referred to the ED by the ambulatory physician contacted.

### Network approach to SRW consultation determinants, features and motives

**Fig. 1:**
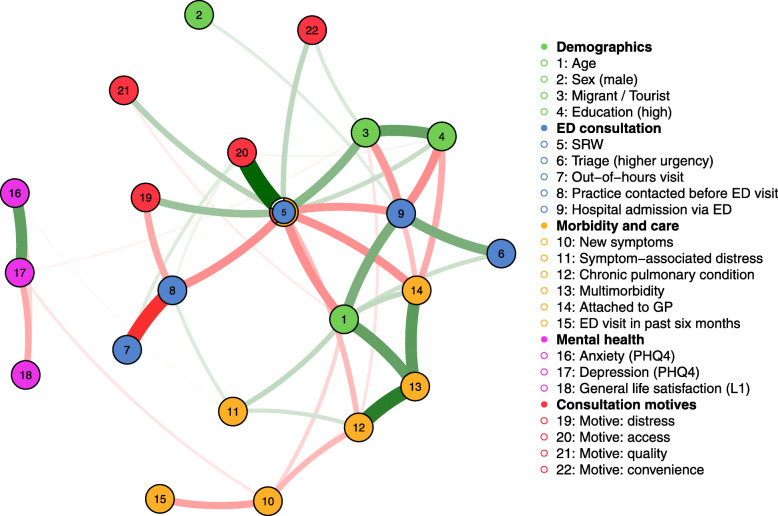
MGM network plot of patient characteristics, consultation motives, ED consultation features and outcomes. *Note*. Green edges indicate positive, red edges negative associations. The ring around the SRW node visualizes predictability of SRW by the remaining network nodes: The orange part indicates the accuracy of the intercept (marginal) model and the red part shows the additional accuracy achieved by connected nodes. As the network graph is force-directed, graphical spacing of two connected nodes does not reliably represent the magnitude of their association, thus barring spatial interpretations [50]. Multi-categorical variables “migration and travel” and “education” were binarized to avoid unsigned edges. For migration, this meant categorization of participants not born in Germany (first-generation migrants and tourists) vs. others; Regarding education, dichotomization threshold was set between CASMIN categories for high education (=tertiary) vs. intermediate and lower education (=primary and secondary)

The estimated MGM network shows that demographic patient characteristics, morbidity and care attributes, consultation features and decision-making in ED utilization are complexly intertwined. In the regularized network (Fig. 1), the SRW node features the highest number of connections (edges) and is positively linked with the four consultation motive groups as well as with a high education level and being a first-generation migrant or tourist. Negative edges are visible between SRW and having contacted a practice prior to the ED visit, hospital admission, age, chronic pulmonary conditions and having a GP. Predictability of SRW in the network was 0.8, with an increase of 0.21 beyond the marginal probabilities.

**Fig. 2:**
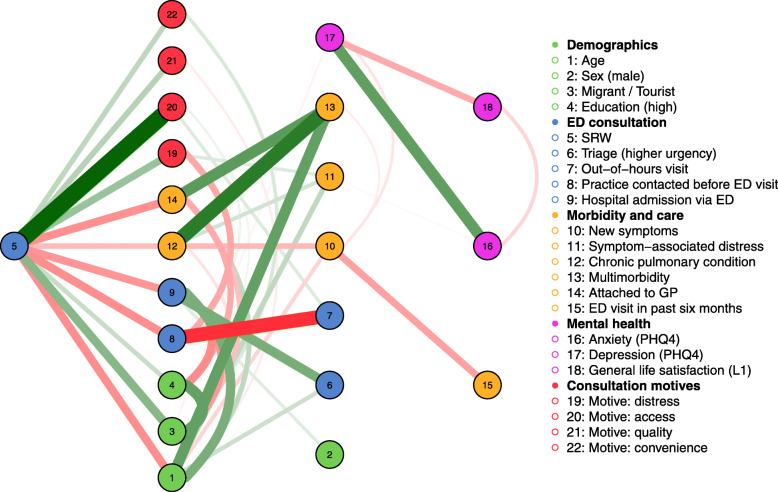
Flow plot of MGM network with SRW as node of interest. *Note.* With the node of interest located on the left, all other nodes are subsequently drawn in vertical levels to the right, in the order of direct connectiveness to the node of interest. This representation shows how one node connects to all other nodes in the network

Connections of SRW in the network correspond to the results of the preceding regression of SRW determinants, with non-zero edges estimated for all predictors identified in the logistic model. A flow plot (Fig. 2) makes the direct connections between SRW and its demographic and medical determinants and underlying motives more easily visible. Notably, characteristics located in the second level (e.g. triage category, multimorbidity) did likewise not show statistical significance as SRW predictors in the logistic model.

Among nodes with direct links to SRW, it strikes that the demographic characteristics (green) and morbidity and care attributes (yellow) show more connections and interconnections than the motives (red). Looking at the few connections between motives and other network nodes, the included edges appear fundamentally plausible, thus for example the edges between “motive: access” and “out-of-hours” and “practice contacted before ED visit”. Beyond this, motives seem comparably self-determined. Except from a few tentative clues (e.g. edge between “migrant/tourist” and “motive convenience”), we cannot link motives to distinct patient groups or characteristics. Predictability of the motive nodes in the network correspondingly does not exceed marginal probabilities (motives “access”, “quality” and “convenience”) or is minimal (“distress”). This is additionally suggestive of motives being mainly influenced by unknown factors not included in the network.

As to auxiliary analyses, bootstrapping showed sizable confidence intervals around edge-weights, suggesting that many weights might not significantly differ; we thus refrained from interpretation of their order. We additionally explored the effects of adding hospital diagnoses to the network, categorized as “potentially more severe” (pneumonia, respiratory insufficiency), “potentially less severe” (upper airway conditions, RTI, or R diagnoses only) and “chronic illness-related” (COPD, asthma). However, predictability of SRW did not improve by inclusion of diagnoses.

## Discussion

### Determinants of SRW consultations

The results suggest that the “road” ultimately leading to an SRW visit starts with certain predisposing demographic traits and medical and care characteristics, like younger age, absence of chronic illness, migration background, and having no regular GP. Corresponding findings of a higher tendency of the young to self-refer [12] and consult non-urgently [3, 7] have been described by others. The same applies to a higher ED utilization by migrant populations, which was reported for most European countries for which evidence was available in a systematic review by Graetz et al. [51]. Alleged reasons for greater migrant utilization encompass health status, cultural factors, as well as care structures in peoples’ countries of origin, including possible experiences of poor-quality PC [51]. In contrast, a population-based study from Germany described a prevalent PC-based healthcare utilization pattern in first-generation migrants and linked this to lower socioeconomic and educational status [52]. In our cohort however, first-generation migrants were comparatively well-educated (tertiary education 47.0% vs. 23.7% in participants with no migration background). This might in part be an age effect (median 42 years, vs. 60 years in people with no migration background), but could also be influenced by the metropolitan setting, with some inner-city hospitals potentially attracting young and internationally mobile professionals not representative of a general migrant population. About 30% of the first-generation migrants in our study population reported to have lived in Germany five years or less, which might support this notion. The countries of birth assessed in the survey do not offer any obvious further clues here, with 26.5% born in EU countries, followed by 14.7% Middle East, 11.8% Turkey and 10.8% Latin America as largest subgroups. Another possibility comes to mind in this context: the result of highly educated immigrants being overrepresented in the study could have been biased by participants’ language skills, as interviews were conducted in German and English only. While the written questionnaire was additionally translated into Turkish and Arabic, these versions had to be answered in writing, requiring reading and writing skills proficient enough to answer a quite extensive set of questions. The lower-threshold option of being interviewed was thus only available to people with a good working knowledge of German or English. This could have led to a selection bias towards better-educated people that could explain part of this result. The high proportion of people with academic education in our cohort corresponds to the Berlin tertiary education rate, which distinctly exceeds the nationwide average [53], a phenomenon underscoring the special circumstances of a big-city location. Studies from other health care contexts have reported a higher tendency to self-refer or consult non-urgently in groups of lower socioeconomic status [54, 55], and our discrepant results may be attributable to setting effects. Concerning interactions between care sectors, the seemingly extenuating effect of having a GP on self-referrals and less urgent ED consultations is in line with other results [56, 57].

### Motives and decision-making

How do patients – whether they feature predisposing characteristics for SRW or not – reach their decision to consult further “down the road”? In contrast to previous studies not centering on a specific trigger symptom [6, 9, 12], our results do not indicate an important role of convenience considerations. The relative preponderance of e.g. distress as a motive could be attributable to the nature of respiratory symptoms, their seriousness vs. benignancy being potentially more difficult to appraise for the patient than for e.g. an injury or rash, thus enhancing subjective urgency [3]. Some patients appear to feel too severely ill to consider alternative care options, and thus are less likely to try to contact a practice, as suggested by the negative network edge between “Practice contacted” and “Motive: distress”. Concurrently, other studies have repeatedly described health concerns and medical necessity as important consultation motives in ED self-referrers [8, 58]. Concerning access problems, the results are in line with previous studies having discussed the important role of PC availability as a determinant of ED utilization [9, 59, 60].

Our data interestingly suggest that the said consultation motives are only sparingly connected with demographic and medical patient characteristics and cannot be attributed to distinct patient groups, so we cannot readily derive “why” from “who”. Motives and decision-making presumably depend on other factors. Speculatively, distress for example might be influenced by individual experiences and personal susceptibility to health-related anxiety, and access problems may depend on the reachability of the individual patient’s GP. However, this remains conjecture, and qualitative methods might constitute a more appropriate approach for studying the role of factors like personality traits, experiences or social environment.

### Urgent and appropriate - or not?

Much has been written about non-urgent ED patients and inappropriate utilization. Unlike others [11, 61], we did not attempt to classify SRW patients as appropriate or inappropriate (or urgent vs. non-urgent), as selecting reasonable criteria is controversial [7]. We would like to stress that we do not consider SRW utilization as congruent with “non-appropriate”: among the SRW crowd, there are a non-negligible proportion of patients with hallmarks of medically serious situations, e.g. pneumonia or respiratory failure diagnoses. Globally however, our study results show that SRW patients are comparatively less severely ill and less likely to be hospitalized. While quantifying the extent of avoidable ED visits was not part of our research question, these observations suggest a substantial share.

### How to intervene?

Our results as well as the literature suggest that having a regular GP has a regularizing effect. Measures to encourage PC attachment could supposedly advance better-targeted utilization. Beyond GP care, the demographic and medical predisposing traits identified inherently seem difficult to influence. Thus, the prevalent problem areas of distress and access stand out as most promising gateways for health care interventions.

The scope of patients’ distress supposedly depends on the self-assessment of symptoms experienced [62]. Besides personality traits, health literacy may affect the capacity for adequate interpretation of bodily sensations; corresponding deficits could contribute to patients seeing no alternative to an ED visit despite not being severely ill [63, 64]. A worthwhile avenue to explore in further studies might be provision of guidance for adequate “self-triage”. Evidence on corresponding online decision-support aids is currently controversial [65], but some approaches seem promising [66].

The effectiveness of measures aimed at ameliorating PC access problems for reducing ED burden remains controversial. The current body of evidence suggests that additional offers like a co-location of GP posts and emergency departments are probably more promising than a simple expansion of regular GPs’ office hours [67]. A combination with information supporting utilization decisions could be worthwhile [68], thus making the connection to the health literacy issue. However, this needs to be further substantiated scientifically, keeping in mind the question of cost-effectiveness of new care structures. In Germany, the government recently has proposed a bill aimed at reforming emergency care structures to antagonize ED crowding [69]. This includes the establishment of special emergency centers at hospitals, where patients will be sent either to the ED or outpatient care structures, depending on severity of illness. These are planned to be operated jointly by the hospitals and the Association of Statutory Health Insurance Physicians. International experiences with similar concepts appear promising: in the Netherlands for example, Emergency Care Access Points (ECAP) jointly created by EDs and GP cooperatives have demonstrated considerable effects on reducing ED consultations [70]. The German plans also encompass fusing the currently separate call centers for EMS and non-emergency out-of-hours doctors. Ensuring adequate reimbursement for EMS care provided on-site is also included in the reform, as currently EMS transport is generally only paid for if patients are brought to hospital. The effects of this proposed package of measures on ED utilization remain to be seem in the coming years.

### Strengths and limitations

This study provides a comprehensive insight into the determinants of SRW consultations for respiratory complaints as well as underlying motives. To our knowledge, it is the first study to explore the complex connections of factors associated with ED utilization by a network method. Network approaches and mixed graphical models have been increasingly applied in the context of clinical psychology and psychometrics [71, 72], but their use for visualizing and studying complex relationships in health services research is novel. The network approach underscores the results of logistic regression by a different modeling method and offers additional insight into the interconnections of variables. However, we were quite careful regarding inference, bootstrapping having revealed limited edge weight stability. Future studies with larger sample sizes might allow more robust estimations here. This limitation however does not apply to the same extent to the presence of edges, as observation of edges not set to zero in a regularized network already indicates that the edge is sufficiently strong to be included in the model [43].

Several additional caveats apply. For once, potential selection bias must be considered. In an ED setting, not all patients may be similarly inclined to participate in a study, depending on factors like e.g. severity of illness – or language skills, as we have already discussed. On the other hand, the inclusion of all hospitals in the city district ensured access to a wide-ranging group of ED patients in a high-density urban area and serves to mitigate selection effects specific to single-center studies [21]. Regarding representativeness, it must be noted that the deferred recruitment of inpatients induces an overrepresentation of less severe cases in our cohort, and certainly SRW cases as well. However, while this influences the relative representation of utilizer groups in the study population, it does not affect interpretability of differences between groups. Furthermore, the study’s focus on respiratory complaints limits generalizability to unselected populations, even if the selected model symptom is frequent and includes a wide spectrum of underlying severe and non-severe constellations. Studies considering all possible diagnoses though pose other problems, e.g. a need to differentiate between medical and surgical indications. Beyond all this, we must emphasize that the study was conducted prior to the advent of COVID-19, which has currently changed the implication of respiratory symptoms dramatically.

Concerning the data collection methods and tools used in our study, we would like to point out that many questionnaire items and scales were newly developed for this study on a theoretical basis, as we could not identify any validated tools (e.g. for assessing symptom-associated distress). Thus, we cannot attest to the sensitivity and specificity of these scales. Neither can we exclude that some questionnaire items might have been interpreted by study participants in a way not intended by us: when inquiring about consultation motives for example, patients might have felt prompted to justify their choice, rather than to just explain it.

As to consultation motives, we would additionally like to stress that quantitative methods can only schematically assess decision-making processes and are ill suited to capture cognitive and emotional goings-on. Qualitative studies have explored such issues in greater depth [6, 9]. Concerning our study, the results of an ancillary GP interview module have been published [20], a paper on the patient perspective is in preparation.

## Conclusions

As to the question of “who” consults in an SRW manner, we identified demographic and medical determinants enhancing corresponding probabilities in respiratory ED patients. The young, well-educated, and pulmonary healthy as well as migrants must be mentioned here. Having a regular GP reduces the chance of SRW utilization. The said characteristics seem only barely connected to “why” people decide on SRW visits. Subjective distress and PC access problems play a pivotal role as consultation motives in the focused population, while convenience seems comparably inconsequential, thus tendentially confuting the notion of irresponsible utilization. Interventions to reduce non-urgent ED use should focus on patients’ ability for symptom self-assessment and care structures alleviating PC access barriers.

## Supplementary information

**Additional file 1.** Fragebogen EMACROSS. Survey questionnaire – German language version.

**Additional file 2.** Questions EMACROSS. Survey questionnaire – English language version.

## Data Availability

The datasets used and analyzed during this study are available from the corresponding author on reasonable request.

## Abbreviations

ED: emergency department
GP: general practitioner
EU: European Union
ECAP: emergency care access point
PC: primary care
ACSC: Ambulatory Care Sensitive Conditions
eCRF: electronic case report form
SRW: self-referred walk-in
EMS: emergency medical services
EPV: events per variable
ROC: receiver operating characteristic
MGM: mixed graphical model
LASSO: least absolute shrinkage and selection operator
EBIC: Extended Bayesian Information Criterion
AUC: area under the curve
ICD: International Classification of Diseases
COPD: chronic obstructive pulmonary disease
RTI: respiratory tract infection
COVID-19: coronavirus disease 2019
