# Supplementary material for: Self-referred walk-in patients in the emergency department – who and why? Consultation determinants in a multicenter study of respiratory patients in Berlin, Germany
Source: BMC Health Serv Res. 2020 Sep 10;20:848. doi: 10.1186/s12913-020-05689-2 (PMC7481545; doi:10.1186/s12913-020-05689-2)
Supplement: Supplementary file 1 — Additional file 1. Fragebogen EMACROSS. Survey questionnaire – German language version. [file 12913_2020_5689_MOESM1_ESM.pdf]

Fragebogen EMACROSS

EMANet
Netzwerk für Versorgungsforschung in der Notfall- und Akutmedizin Berlin

Pseudonym (EMAxy_Zentrum_00xy): Datum der Befragung:

**1. Welches Geschlecht haben Sie?**	
Männlich	☐
Weiblich	☐
**2. Wann sind Sie geboren? Bitte geben Sie den <u>Monat</u> und das <u>Jahr</u> Ihrer Geburt an.**	
___________________________ (MM/JJJJ)	
Weiß nicht / keine Angabe	☐
**3. Welche Hauptbeschwerden sind aus Ihrer Sicht der Grund für Ihren heutigen Notaufnahmebesuch?** *Sie können mehrere Antworten ankreuzen.*	
Husten	☐
Atemnot	☐
Auswurf	☐
Fieber	☐
Schnupfen	☐
Halsschmerzen	☐
Schmerzen im Thorax	☐
Ohrenschmerzen	☐
Kopfschmerzen	☐
Müdigkeit / Erschöpfung	☐
Gliederschmerzen	☐
Schwindel	☐
Übelkeit / Erbrechen	☐
Weiß nicht / keine Angabe	☐
Sonstige Beschwerden: ____	☐

**4. Seit wann treten die aktuellen Beschwerden auf?**<br>*Bitte nur eine Antwort ankreuzen.*	
Seit dem heutigen Tag	☐
Seit dem gestrigen Tag	☐
Seit einigen Tagen (aber nicht länger als 1 Woche)	☐
Seit mehr als einer Woche	☐
Weiß nicht / keine Angabe	☐
**5. Wie häufig treten Ihre aktuellen Beschwerden auf?**<br>*Bitte nur eine Antwort ankreuzen.*	
Zum ersten Mal aufgetreten	☐
Einmal vorher aufgetreten	☐
Treten immer wieder auf	☐
Liegen dauerhaft vor	☐
Weiß nicht / keine Angabe	☐
**6. Haben Sie hier in der Notaufnahme bereits Informationen über das Untersuchungs- und Behandlungsergebnis erhalten?**	
Ja	☐
Nein	☐
Weiß nicht / keine Angabe	☐
**7. Welche Erkrankung oder Diagnose ist aus Ihrer persönlichen Sicht die Ursache Ihrer aktuellen Beschwerden?**<br>*Sie können mehrere Antworten ankreuzen.*	
Grippaler Infekt / Erkältungskrankheit	☐
Akute Bronchitis	☐
Grippe / Influenza	☐
Asthma / Asthmaanfall	☐

COPD / chronische Bronchitis / Lungenemphysem	☐
Lungenentzündung	☐
Weiß nicht / keine Angabe	☐
Sonstige Erkrankungen: ____	☐
**8. Wie schwerwiegend sind die aktuellen Beschwerden für Sie auf einer Skala von 0 bis 10?**	
Überhaupt nicht schwerwiegend — Extrem schwerwiegend<br>o---------o---------o--------o---------o---------o---------o---------o--------o---------o---------o<br>0 1 2 3 4 5 6 7 8 9 10	
Weiß nicht / Keine Angabe	☐
**9. Wie dringlich schätzen Sie persönlich Ihren Behandlungsbedarf ein?**<br>*Bitte nur eine Antwort ankreuzen.*	
Akut (Ich muss sofort vom Arzt gesehen werden)	☐
Sehr dringlich (Ich muss so schnell wie möglich vom Arzt gesehen werden)	☐
Dringlich (Ich muss noch heute vom Arzt gesehen werden)	☐
Weniger dringlich (aber ich selbst mache mir Sorgen bzw. andere sorgen sich um mich)	☐
Weiß nicht / keine Angabe	☐
**10. Wie bedrohlich schätzen Sie die aktuelle Notfallsituation auf einer Skala von 0 bis 10 ein?**	
Überhaupt nicht bedrohlich — Extrem bedrohlich<br>o---------o---------o--------o---------o---------o---------o---------o--------o---------o---------o<br>0 1 2 3 4 5 6 7 8 9 10	
Weiß nicht / Keine Angabe	☐

11. Wie oft fühlten Sie sich im Verlauf der letzten 2 Wochen durch die folgenden Beschwerden beeinträchtigt?

0 = Überhaupt nicht, 1 = An einzelnen Tagen, 2 = An mehr als der Hälfte der Tage, 3 = Beinahe jeden Tag

Wenig Interesse oder Freude an Ihren Tätigkeiten

	0 ☐	1 ☐	2 ☐	3 ☐

Niedergeschlagenheit, Schwermut oder Hoffnungslosigkeit				
	0 ☐	1 ☐	2 ☐	3 ☐
Nervosität, Ängstlichkeit oder Anspannung				
	0 ☐	1 ☐	2 ☐	3 ☐
Nicht in der Lage sein, Sorgen zu stoppen oder zu kontrollieren				
	0 ☐	1 ☐	2 ☐	3 ☐

12. Haben Sie eine oder mehrere lang andauernde, chronische Krankheiten (Chronische Krankheiten sind lang andauernde Erkrankungen, die ständiger Behandlung und Kontrolle bedürfen) – und wenn ja, welche?

Ja	☐
Nein	☐
Weiß nicht / keine Angabe	☐

Wenn Sie eine unter einer chronischen Erkrankung leiden, kreuzen Sie bitte die entsprechende Erkrankung an oder notieren Sie diese unten im Freitextfeld. Sie können auch mehrere Erkrankungen angeben.

**Herz-Kreislauf und Stoffwechsel, z.B. Bluthochdruck, erhöhte Blutfette, Diabetes**	☐
Bluthochdruck	☐
Erhöhte Blutfette	☐
Diabetes mellitus	☐
Koronare Herzkrankheit, z.B. Angina pectoris, Herzinfarkt (jemals gehabt)	☐
Herzinsuffizienz	☐
Schlaganfall (jemals gehabt)	☐
**Lungenerkrankungen, z.B. chronische Bronchitis, Asthma**	☐
Asthma	☐
Chronische Bronchitis	☐
**Leber- und Nierenerkrankungen**	☐
Nierenerkrankung	☐

Lebererkrankung	☐
**Verdauungssystem, z.B. Magen- oder Darmgeschwür, Schleimhautentzündung, Morbus Crohn**	☐
Entzündung oder Geschwür in Magen oder Zwölffingerdarm	☐
Morbus Crohn, Colitis ulcerosa	☐
**Bewegungsapparat, z.B. Rheuma, Arthrose/Gelenkverschleiß, chronische Rückenschmerzen**	☐
Arthrose / Gelenkverschleiß	☐
Rheumatische Erkrankung	☐
Osteoporose	☐
Chronischer Rückenschmerz oder sonstiger chronischer Schmerz	☐
**Krebs (jemals gehabt)**	☐
**Psychische Erkrankungen, z.B. Depression, Ängste**	☐
Depression	☐
Angststörung	☐
**Sinnesorgane, z.B. schwere Einschränkung des Seh- oder Hörvermögens, grauer/grüner Star**	☐
Schwere Einschränkung des Sehvermögens	☐
Schwere Einschränkung des Hörvermögens	☐
Grauer Star (Katarakt)	☐
Grüner Star (Glaukom)	☐
**Hormon- und Immunsystem, z.B. Schilddrüsenerkrankung, Autoimmunerkrankung**	☐
Schilddrüsenüber- oder -unterfunktion	☐
Autoimmunerkrankung	☐
**Hauterkrankung, z.B. Schuppenflechte, Neurodermitis**	☐
Schuppenflechte (Psoriasis)	☐

Neurodermitis	☐
**Nervenssystem, z.B. Parkinson, Multiple Sklerose, Epilepsie**	☐
Morbus Parkinson	☐
Multiple Sklerose	☐
Epilepsie	☐
**Urogenitalsystem**	☐
Blasenschwäche	☐
**Sonstige körperliche oder psychische Erkrankung(en):** ____	☐
**13. Sind Sie zurzeit oder waren Sie in den vergangenen 6 Monaten krankgeschrieben?**	
Ja	☐
Nein	☐
Weiß nicht / keine Angabe	☐
Folgende Frage nur beantworten, wenn Sie die vorherige Frage mit „Ja" beantwortet haben. **13A. Wenn ja, wie lange waren Sie in den letzten 6 Monaten insgesamt krankgeschrieben? Bitte geben Sie die Krankheitstage in Wochentagen an.**	
Krankheitstage: ___________ (in Wochentagen)	
Weiß nicht / keine Angabe	☐
**14. Haben Sie einen Hausarzt?**	
Ja	☐
Nein	☐
Weiß nicht / keine Angabe	☐
**15. Welche der nachfolgenden Ärzte und medizinischen Leistungen haben Sie in den letzten 6 Monaten in Anspruch genommen und wie häufig (Hausbesuche mitgerechnet)?** *Es sind bei Arztbesuchen nur Termine gemeint, bei denen Sie den Arzt persönlich gesprochen haben – der aktuelle Besuch in der Notaufnahme zählt nicht dazu.*	

Sie können mehrere Antworten ankreuzen. Bitte geben Sie auch an, wie häufig Sie den Arzt / die medizinische Leistung in Anspruch genommen haben.

			**Wie häufig?**
Hausarzt	Ja	☐	_______ Mal
	Nein	☐	
	Weiß nicht / keine Angabe	☐	
Ambulanter Facharzt	Ja	☐	_______ Mal
	Nein	☐	
	Weiß nicht / keine Angabe	☐	
Lungenfacharzt	Ja	☐	_______ Mal
	Nein	☐	
	Weiß nicht / keine Angabe	☐	
Notaufnahme (außer heute)	Ja	☐	_______ Mal
	Nein	☐	
	Weiß nicht / keine Angabe	☐	
Stationärer Krankenhausaufenthalt	Ja	☐	**Wie lange?** Gesamte Aufenthaltsdauer aller Aufenthalte in Tagen: _____________
	Nein	☐	
	Weiß nicht / keine Angabe	☐	

15A. Falls Sie in den letzten 6 Monaten in einer Notaufnahme waren:

Diese Frage bitte nur beantworten, wenn Sie in den letzten 6 Monaten in einer Notaufnahme waren - den heutigen Besuch in der Notaufnahme nicht mitgerechnet. Ansonsten bitte mit der nächsten Frage „Haben Sie in den letzten 6 Monaten den Hausbesuchs-Notdienst…“ weitermachen.

Wie viele verschiedene Notaufnahmen haben Sie in den letzten 6 Monaten	Anzahl: _________________

aufgesucht? (den heutigen Notaufnahmebesuch nicht mitgezählt)	
Wegen welcher Beschwerden?	☐ Gleiche oder ähnliche Beschwerden wie aktuell
	Andere Beschwerden, nämlich ________________
Weiß nicht / keine Angabe	☐

**16. Haben Sie in den <u>letzten 6 Monaten</u> den Hausbesuchs-Notdienst der Kassenärztlichen Vereinigung Anspruch genommen (ärztlicher Bereitschaftsdienst „rosa Auto“) – und wenn ja, wie häufig?**	
Ja	☐
Wenn ja, wie oft?	_______ Mal
Nein	☐
Weiß nicht / keine Angabe	☐
Die folgenden drei Fragen zum Hausarzt bitte nur beantworten, wenn Sie einen Hausarzt haben. Wenn Sie keinen Hausarzt haben, bitte bei Frage Nr. 20 "Wer hat die Entscheidung getroffen…" auf S. 10 weitermachen.	
**17. Seit wie vielen Jahren sind Sie Patient bei Ihrem Hausarzt?** *Bitte nur eine Antwort ankreuzen.*	
Bis zu einem Jahr	☐
Über ein bis fünf Jahre	☐
Über fünf bis zehn Jahre	☐
Über 10 Jahre	☐
Weiß nicht / keine Angabe	☐
**18. Wie zufrieden sind Sie insgesamt mit der Betreuung durch Ihren Hausarzt?** *Bitte nur eine Antwort ankreuzen.*	
Sehr zufrieden	☐
Zufrieden	☐
Weder zufrieden noch unzufrieden	☐
Unzufrieden	☐
Sehr unzufrieden	☐

Weiß nicht / keine Angabe						☐
**19. Die folgenden Aussagen treffen auf meinen Hausarzt zu:**						
Ich kenne meinen Hausarzt sehr gut						
	☐	☐	☐	☐	☐	☐
	Stimme voll zu	Stimme zu	Neutral	Stimme nicht zu	Stimme gar nicht zu	Weiß nicht / Keine Angabe
Mein Hausarzt kennt meine medizinische Vorgeschichte sehr gut						
	☐	☐	☐	☐	☐	☐
	Stimme voll zu	Stimme zu	Neutral	Stimme nicht zu	Stimme gar nicht zu	Weiß nicht / Keine Angabe
Mein Hausarzt weiß immer sehr gut Bescheid, was er bisher gemacht hat						
	☐	☐	☐	☐	☐	☐
	Stimme voll zu	Stimme zu	Neutral	Stimme nicht zu	Stimme gar nicht zu	Weiß nicht / Keine Angabe
Mein Hausarzt kennt meine Familiensituation sehr gut						
	☐	☐	☐	☐	☐	☐
	Stimme voll zu	Stimme zu	Neutral	Stimme nicht zu	Stimme gar nicht zu	Weiß nicht / Keine Angabe
Mein Hausarzt weiß sehr gut über mein tägliches Leben Bescheid						
	☐	☐	☐	☐	☐	☐
	Stimme voll zu	Stimme zu	Neutral	Stimme nicht zu	Stimme gar nicht zu	Weiß nicht / Keine Angabe
Mein Hausarzt kontaktiert mich wenn notwendig, ohne dass ich nachfragen muss						
	☐	☐	☐	☐	☐	☐
	Stimme voll zu	Stimme zu	Neutral	Stimme nicht zu	Stimme gar nicht zu	Weiß nicht / Keine Angabe
Mein Hausarzt weiß sehr gut darüber Bescheid, was mir in meiner medizinischen Versorgung wichtig ist						

	☐	☐	☐	☐	☐	☐
	Stimme voll zu	Stimme zu	Neutral	Stimme nicht zu	Stimme gar nicht zu	Weiß nicht / Keine Angabe
Mein Hausarzt bleibt mit mir in Kontakt, wenn ich von Anderen behandelt werde						
	☐	☐	☐	☐	☐	☐
	Stimme voll zu	Stimme zu	Neutral	Stimme nicht zu	Stimme gar nicht zu	Weiß nicht / Keine Angabe

**20. Wer hat die Entscheidung getroffen, dass Sie in die Notaufnahme kommen sollten?** *Bitte nur eine Antwort ankreuzen.*	
Habe mich selbst zum Besuch entschieden	☐
Passanten / Fremde	☐
Angehörige, Freunde, Bekannte, Arbeitskollegen, Vorgesetzte	☐
Hausarzt	☐
Anderer ambulanter Arzt	☐
Ein anderes Krankenhaus hat mich verwiesen bzw. weitergeleitet	☐
Eine Pflegekraft (ambulant oder stationär)	☐
Anderes: ____	☐
Weiß nicht / keine Angabe	☐
**21. Wie sind Sie in die Notaufnahme gekommen?** *Bitte nur eine Antwort ankreuzen.*	
Ich bin mit Rettungsdienst / Krankenwagen / Notarzt / Feuerwehr gekommen	☐
Ich wurde von Angehörigen / Freunden gebracht	☐
Ich bin selbst gefahren (Mit Motorfahrzeug)	☐
Ich bin zu Fuß gekommen	☐
Ich bin mit öffentlichen Verkehrsmitteln gekommen	☐
Ich bin mit dem Taxi gekommen	☐

Weiß nicht / keine Angabe	☐
**22. Haben Sie vor Ihrem Besuch in der Notaufnahme versucht, Kontakt zu einer Arztpraxis aufzunehmen?**	
Ja, Hausarzt	☐
Ja, Facharzt	☐
Nein	☐
Weiß nicht / keine Angabe	☐
**23. Warum haben Sie sich entschieden, mit Ihren Beschwerden in eine Notaufnahme zu kommen?**<br>*Sie können mehrere Antworten ankreuzen.*	
Weil mich der Rettungsdienst / Notarzt gebracht hat bzw. ein Arzt eingewiesen hat	☐
Weil die Beschwerden so stark waren	☐
Weil die Situation für mich bedrohlich war / weil ich Angst hatte	☐
Weil mein Hausarzt derzeit nicht geöffnet hat:<br>☐ Nacht<br>☐ Wochenende / Feiertag<br>☐ Urlaub<br>☐ Außerhalb der Sprechzeiten werktags	☐
Weil ich meinen Hausarzt / Facharzt nicht erreicht habe<br>☐ Hausarzt<br>☐ Facharzt	☐
Weil ich keine Zeit habe, wenn die Arztpraxen geöffnet sind (z. B. wegen der Arbeit)	☐
Weil ich, obwohl ich es versucht hatte, keinen schnellen Termin bekommen habe beim (Mehrfachnennung möglich)<br>☐ Hausarzt<br>☐ Facharzt	☐
Weil ich nicht so lange warten wollte wie in einer Arztpraxis (Wartezeit im Wartebereich)	☐
Weil ich hier im Krankenhaus besser behandelt werde als in einer Arztpraxis	☐
Weil die Abläufe im Krankenhaus gut organisiert sind	☐
Weil im Krankenhaus mehr Untersuchungen (Labor, Röntgen usw.) gemacht werden können bzw. die Untersuchung / Behandlung umfassender ist	☐

Weil ich eine zweite Meinung haben wollte	☐
Weil ich in dieser Stadt nur zu Besuch bin	☐
Weil ich erst seit kurzem in Berlin wohne und keinen Arzt kenne	☐
Weil die Diagnostik- / Untersuchungsergebnisse schnell verfügbar sind	☐
Weil im Krankenhaus besondere Spezialisten arbeiten	☐
Weil die Notaufnahme immer offen ist und man keinen Termin braucht	☐
Weil ich zur Notaufnahme von meiner Wohnung nicht so einen weiten Weg habe wie zum niedergelassenen Arzt	☐
Weil die Notaufnahme besser erreichbar ist als eine Arztpraxis (Parkplätze, ÖPNV-Anbindung)	☐
Weil ich bei vorherigen Besuchen in der Notaufnahme zufrieden mit der Behandlung war	☐
Weiß nicht / Keine Angabe	☐
Andere Gründe: ____	☐
**24. Hätte aus Ihrer Sicht auch ein Hausarzt Ihr Problem lösen können?**	
Ja	☐
Nein	☐
Weiß nicht / keine Angabe	☐
**25. Warum sind Sie gerade in diese Notaufnahme gekommen?**<br>*Sie können mehrere Antworten ankreuzen.*	
Hat der Rettungsdienst / Notarzt / einweisende Arzt so entschieden bzw. empfohlen	☐
Verkehrsmäßig gut erreichbar	☐
Nahe an meiner Wohnung / Arbeit	☐
Diese Notaufnahme / dieses Krankenhaus hat einen guten Ruf	☐
Spezialisierte Ärzte / Abteilungen vorhanden	☐
Spezielle diagnostische Verfahren verfügbar	☐
Ich war schon einmal hier und bin hier bekannt	☐

Ich war schon einmal hier und war mit der Behandlung zufrieden	☐
Weiß nicht / keine Angabe	☐
Andere Gründe:____	☐
**26. Wie zufrieden sind Sie mit Ihrer heutigen Behandlung in der Notaufnahme?** *Bitte nur eine Antwort ankreuzen.*	
Sehr zufrieden	☐
Zufrieden	☐
Weder zufrieden noch unzufrieden	☐
Unzufrieden	☐
Sehr unzufrieden	☐
Weiß nicht / keine Angabe	☐
**27. Wenn Sie nicht zufrieden sind mit Ihrer heutigen Behandlung in der Notaufnahme: Was sind die Gründe dafür, dass Sie nicht zufrieden sind?** *Sie können mehrere Antworten ankreuzen.*	
Meine Beschwerden wurden nicht ernstgenommen	☐
Man hätte mich stationär aufnehmen müssen	☐
Die Wartezeiten waren zu lang	☐
Die Abläufe in der Notaufnahme sind schlecht organisiert	☐
Das Personal war unfreundlich	☐
Es wurden unnötige Untersuchungen durchgeführt	☐
Es wurden wichtige Untersuchungen nicht durchgeführt	☐
Ich glaube nicht an die Richtigkeit der hier gestellten Diagnose	☐
Ich habe keine ausreichende Behandlung meiner akuten Beschwerden / Schmerzen erhalten	☐
Es wurden unnötige Behandlungen durchgeführt	☐
Ich habe keine oder zu wenige Medikamente für die Zeit nach der Entlassung erhalten	☐

Ich habe nicht genug Information (über meine Behandlung, den weiteren Verlauf etc.) erhalten	☐
Ich konnte nicht genug mit meinen Angehörigen etc. kommunizieren	☐
Ich konnte mit dem Personal aufgrund von Sprachproblemen nicht ausreichend kommunizieren	☐
Weiß nicht / keine Angabe	☐
Andere Gründe :____	☐
**28. Was machen Sie, wenn Sie aus der Notaufnahme entlassen werden?**<br>*Bitte nur eine Antwort ankreuzen.*	
Ich werde nichts weiter unternehmen	☐
Ich werde zu meinem Hausarzt gehen, um mich weiter untersuchen oder behandeln zu lassen	☐
Ich werde zu einem Facharzt gehen, um mich weiter untersuchen oder behandeln zu lassen	☐
Das kann ich noch nicht sagen, da die Behandlung hier noch nicht abgeschlossen ist	☐
Sonstiges: ____	☐
Weiß nicht / keine Angabe	☐
**Sie haben einen Großteil des Fragebogens bereits beantwortet. Im Folgenden möchten wir Ihnen nun noch einige Fragen zu Ihrer Person und Lebenssituation stellen.**	
**29. In welchem Land sind Ihre Eltern geboren?**	
**Meine <u>Mutter</u> ist…**	
in Deutschland geboren	☐
in einem anderen Land geboren	☐
Falls Ihre Mutter in einem anderen Land geboren ist, in welchem anderen Land:<br>____	
Weiß nicht / keine Angabe	☐
**Mein <u>Vater</u> ist…**	

in Deutschland geboren	☐
in einem anderen Land geboren	☐
Falls Ihr Vater in einem anderen Land geboren ist, in welchem anderen Land: ____	
Weiß nicht / keine Angabe	☐
**30. Sind Sie in Deutschland geboren?**	
Ja	☐
Nein	☐
Falls Sie in einem anderen Land geboren sind, in welchem anderen Land: ____	
Weiß nicht / keine Angabe	☐
**30A. Falls Sie nicht in Deutschland geboren sind: seit wann leben Sie hauptsächlich in Deutschland? Bitte geben Sie das Jahr an, in dem Sie nach Deutschland gekommen sind.**	
Seit (Jahr): ______________________________	
Weiß nicht / keine Angabe	☐
**31. Welchen <u>höchsten</u> Schulabschluss haben Sie?**	
Volks- oder Hauptschulabschluss; 8. Klasse Polytechnische Oberschule (POS)	☐
Realschulabschluss; 10. Klasse POS	☐
Abitur, Fachhochschulreife / Fachabitur	☐
Noch kein Schulabschluss / noch in der Schule	☐
Kein Schulabschluss	☐
Anderer Schulabschluss, z. B. in einem anderen Land erworben	☐
Weiß nicht / keine Angabe	☐
**31A. Wie viele Jahre haben Sie die Schule besucht?**	

Dauer des Schulbesuchs (in Jahren): ___________________________________	
Weiß nicht / keine Angabe	☐
**32. Welchen <u>höchsten</u> beruflichen Abschluss haben Sie?**	
Berufsbildender Abschluss (Lehre, Berufsaufbauschule, Berufsfachschule / Handelsschule, Fachschule / Meister- oder Technikerschule / einer Schule des Gesundheitswesens)	☐
Akademischer Abschluss (Fachhochschule / Berufsakademie / Ingenieurschule, Hochschule, Universität, Promotion)	☐
Noch in beruflicher Ausbildung (auch Berufsvorbereitungsjahr, Praktikum)	☐
Noch im Studium (auch studienbegleitendes Praktikum, Pflichtpraktikum)	☐
Kein Berufsabschluss	☐
Anderer Berufsabschluss, z. B. in einem anderen Land erworben: ____	☐
Weiß nicht / keine Angabe	☐
**33. Welche der folgenden Angaben trifft auf Ihre derzeitige Erwerbssituation zu?**	
Arbeitnehmer/in	☐
Selbstständige/r	☐
Beurlaubt (z. B. Mutterschutz, Erziehungsurlaub, Elternzeit)	☐
Rentner/in	☐
Arbeitslos	☐
Dauerhaft erwerbsunfähig	☐
Hausfrau / Hausmann	☐
Student/in	☐
Anderes: ____	☐

Weiß nicht / keine Angabe	☐
**34. Wie viele Stunden arbeiten Sie normalerweise insgesamt pro Woche? Bitte geben Sie nur die bezahlten Arbeitsstunden an.**	
Arbeitsstunden / Woche insgesamt (z. B. 37,5 Stunden): ___ , ___ Stunden	
Weiß nicht / keine Angabe	☐
**35. Wo wohnen Sie zurzeit?**	
In einer eigenen Wohnung oder eigenem Haus (Eigentum, zur Miete oder bei Verwandten)	☐
Im betreuten Wohnen (z.B. Alters-WG, Altersheime, Seniorenresidenzen, seniorengerechtes Wohnen)	☐
Stationäre Pflege	☐
Kein fester Wohnsitz	☐
Flüchtlingsunterkunft	☐
Anderes: ____________________________________	☐
Weiß nicht / keine Angabe	☐
**36. Wie viele Personen leben gemeinsam in Ihrem Haushalt?** Zählen Sie bitte sich selbst, (Ehe-)Partner/in, Kinder und Mitbewohner einer Wohngemeinschaft mit.	
1 Person, d. h. nur Sie selbst	☐
Insgesamt ________ Personen	
Weiß nicht / keine Angabe	☐
**37. Wie viele Personen stehen Ihnen so nahe, dass Sie sich bei schweren persönlichen Problemen auf sie verlassen können?**	
Keine	☐
1 oder 2 Personen	☐
3 bis 5 Personen	☐

Mehr als 5 Personen	☐
Weiß nicht / keine Angabe	☐
**38. Welchen Familienstand haben Sie? Was in dieser Liste trifft auf Sie zu?**	
Verheiratet und lebe mit meinem/meiner Ehepartner/-in zusammen	☐
Verheiratet und lebe von meinem/meiner Ehepartner/-in getrennt	☐
Ledig (noch nie verheiratet gewesen)	☐
Geschieden	☐
Verwitwet bzw. feste/r Lebenspartner/-in verstorben	☐
Weiß nicht / keine Angabe	☐
**39. Haben Sie einen festen Partner?**	
Ja	☐
Nein	☐
Weiß nicht / keine Angabe	☐
**Nun möchten wir Ihnen ein paar Fragen zur Gesundheit und Pflege stellen.**	
**40. Sind Sie derzeit in eine Pflegestufe / in einen Pflegegrad eingruppiert?**	
Ja	☐
Nein	☐
Weiß nicht / keine Angabe	☐
**40A. Wenn ja, welche Pflegestufe bzw. welchen Pflegegrad haben Sie? Bitte tragen Sie die Pflegestufe oder den Pflegegrad ein, den Sie haben.**	
1a Pflege**stufe** (1-3): ___ ___	
1b Pflege**grad** (1-5): ___ ___	
Weiß nicht / keine Angabe	☐

**41. Rauchen Sie aktuell?**	
Ja	☐
Nein	☐
Ehemaliger Raucher	☐
Weiß nicht / keine Angabe	☐

**41A. Wie viel wiegen Sie?**	______ kg
**41B. Wie groß sind Sie?**	______ cm
Weiß nicht / keine Angabe	☐

42. Nun würde uns Ihr allgemeiner Gesundheitszustand interessieren.

Diese Skala ist mit Zahlen von 0 bis 100 versehen.

100 ist die beste Gesundheit, die Sie sich vorstellen können. 0 (Null) ist die schlechteste Gesundheit, die Sie sich vorstellen können.

Bitte kreuzen Sie den Punkt auf der Skala an, der Ihre Gesundheit HEUTE am besten beschreibt.

Jetzt tragen Sie bitte die Zahl, die Sie auf der Skala angekreuzt haben, unten ein.

IHRE GESUNDHEIT HEUTE = __________

Beste Gesundheit, die sie sich vorstellen können.

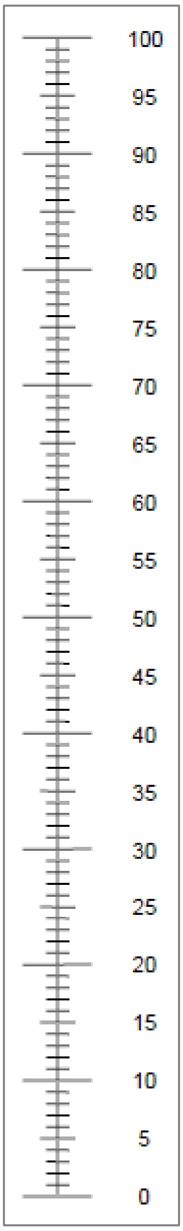

Schlechteste Gesundheit, die Sie sich vorstellen können.

**43. Zudem interessiert uns, wie zufrieden Sie mit Ihrem Leben sind.**<br><br>**Wie zufrieden sind Sie gegenwärtig, alles in allem, mit Ihrem Leben? Bitte kennzeichnen Sie auf der Skala, wie zufrieden Sie sind (0= überhaupt nicht zufrieden, 10= völlig zufrieden)**	
Überhaupt nicht zufrieden Völlig zufrieden<br><br>o---------o---------o--------o---------o---------o---------o---------o--------o---------o---------o<br>0 1 2 3 4 5 6 7 8 9 10	
Weiß nicht / keine Angabe	☐
