# Supplementary material for: Self-referred walk-in patients in the emergency department – who and why? Consultation determinants in a multicenter study of respiratory patients in Berlin, Germany
Source: BMC Health Serv Res. 2020 Sep 10;20:848. doi: 10.1186/s12913-020-05689-2 (PMC7481545; doi:10.1186/s12913-020-05689-2)
Supplement: Supplementary file 2 — Additional file 2. Questions EMACROSS. Survey questionnaire – English language version. [file 12913_2020_5689_MOESM2_ESM.pdf]

Questions EMACROSS

EMANet
Netzwerk für Versorgungsforschung in der Notfall- und Akutmedizin Berlin

Pseudonym (EMAxy_Zentrum_00xy): Date of interview:

**1. What is your sex?**	
Male	☐
Femal	☐
**2. When were you born? Please provide <u>month</u> and <u>year</u> of birth.**	
__________________________ (MM/YYYY)	
I don't know / prefer not to say	☐
**3. From your perspective, what main complaints are the reason for your visit to the emergency department?** *You can select multiple answers.*	
Cough	☐
Shortness of breath	☐
Phlegm / expectoration	☐
Fever	☐
Runny nose / rhinits	☐
Sore throat	☐
Chest pain	☐
Earache	☐
Headache	☐
Tiredness / fatigue	☐
Limb pain	☐
Vertigo / dizziness	☐
Nausea / vomiting	☐
I don't know / prefer not to say	☐
Other complaints: ____	☐

**4. Since when do your current complaints occur?**<br>*Please select only one answer.*	
Since today	☐
Since yesterday	☐
Since a few days ago (but not longer than 1 week)	☐
Since more than 1 week	☐
I don’t know / prefer not to say	☐
**5. How often do these complaints occur?**<br>*Please select only one answer.*	
They occured for the first time	☐
They have occured once before	☐
They occur repeatedly	☐
They are permanently present	☐
I don’t know / prefer not to say	☐
**6. Have you already received any information on the results of your examination and / or treatment here in the emergency department?**	
Yes	☐
No	☐
I don’t know / prefer not to say	☐
**7. From your perspective, what disease or diagnosis is causing your current complaints?**<br>*You can select multiple answers.*	
Common cold	☐
Acute bronchitis	☐
Influenza	☐
Asthma	☐
Chronic obstructive pulmonary disease / chronic bronchitis / emphysema	☐

Pneumonia	☐
I don't know / prefer not to say	☐
Other diseases: ____	☐

8. How severe are your current complaints for you on a scale from 0 to 10?

Not severe at all — Extremely severe

o---------o---------o--------o---------o---------o---------o---------o--------o---------o---------o

0 1 2 3 4 5 6 7 8 9 10

I don't know / prefer not to say	☐

9. From your perspective, how urgently do you need to be treated?

Please select only one answer.

Highly urgent (I have to be seen by the doctor immediately)	☐
Very urgent (I have to be seen by the doctor as soon as possible)	☐
Urgent (I have to be seen by the doctor today)	☐
Less urgent (but I am concerned, or other people are concerned about me)	☐
I don't know / prefer not to say	☐

10. How threatening is the current emergency situation for you on a scale from 0 to 10?

Not threatening at all — Extremely threatening

o---------o---------o--------o---------o---------o---------o---------o--------o---------o---------o

0 1 2 3 4 5 6 7 8 9 10

I don't know / prefer not to say	☐

11. Over the past 2 weeks, have you been bothered by these problems?

0 = Not at all, 1 = Several days, 2 = More days than not, 3 = Nearly every day

Little interest or pleasure in doing things				
	0 ☐	1 ☐	2 ☐	3 ☐
Feeling down, depressed, or hopeless				

	0 ☐	1 ☐	2 ☐	3 ☐
Feeling nervous, anxious, or on edge				
	0 ☐	1 ☐	2 ☐	3 ☐
Not being able to stop or control worrying				
	0 ☐	1 ☐	2 ☐	3 ☐

**12. Do you suffer from one or more long-term, chronic diseases (chronic diseases are usually present for a long time and require continuous treatment and control) - and if yes, which ones?**	
Yes	☐
No	☐
I don't know / prefer not to say	☐
If you suffer from a chronic disease, please select the respective box in the list below or write it down in the free space at the end of the list. You can select multiple diseases.	
**Heart, circulatory system and metabolism, e.g. high blood pressure / hypertension, elevated blood lipids, diabetes**	☐
High blood pressure / hypertension	☐
Elevated blood lipids	☐
Diabetes mellitus	☐
Coronary heart disease, e.g. angina pectoris, myocardial infarction (ever had)	☐
Congestive heart failure	☐
Stroke (ever had)	☐
**Lung diseases, e.g. chronic bronchitis, asthma**	☐
Asthma	☐
Chronic bronchitis	☐
**Liver or kidney disease**	☐
Kidney disease	☐
Liver disease	☐

**Diseases of the digestive tract, e.g. stomach or bowel ulcer, inflammation, Crohn's disease**	☐
Inflammation (e.g. gastritis) in stomach or duodenum	☐
Crohn's disease, ulcerative colitis	☐
**Musculoskeletal system, e.g. rheumatic illness, arthrosis / degenerative joint disease, chronic back pain**	☐
Arthrosis / degenerative joint disease	☐
Rheumatic disease	☐
Osteoporosis	☐
Chronic back pain or other chronic pain syndrome	☐
**Cancer (ever had)**	☐
**Mental illness, e.g. depression, anxiety**	☐
Depression	☐
Anxiety disorder	☐
**Disorders of the sensory organs, e.g. severe impairment of vision or hearing, cataract, glaucoma**	☐
Severe impairment of vision	☐
Severe impairment of hearing	☐
Cataract	☐
Glaucoma	☐
**Endocrine (hormonal) and immune system, e.g. thyroid disease, autoimmune disease**	☐
Hyper- or hypothyroidism	☐
Autoimmune disease	☐
**Skin disease, e.g. psoriasis, atopic dermatitis**	☐
Psoriasis	☐
Atopic dermatitis	☐

**Nervous system, e.g. Parkinson's disease, Multiple sclerosis, epilepsy**	☐
Parkinson's disease	☐
Multiple sclerosis	☐
Epilepsy	☐
**Urogenital system**	☐
Bladder weakness	☐
**Other physical or mental illness** ____	☐
**13. Are you currently on a sick leave or have you been <u>within the last 6 months</u>?**	
Yes	☐
No	☐
I don't know / prefer not to say	☐
Please answer this question only if your answer to the previous question was „yes". **13A. If yes, how long were you on sick leave <u>within the last 6 months</u>? Please provide the number of weekdays on sick leave.**	
Number of days on sick leave / with sick certificate: ___________ (in weekdays)	
I don't know / prefer not to say	☐
**14. Do you have a Family Doctor / General Practitioner?**	
Yes	☐
No	☐
I don't know / prefer not to say	☐
**15. Which of the following doctors / physicians did you visit, or which medical services did you use <u>within the last 6 months</u>, and how often? (please include house calls / home visits of doctors)** *Please count only consultations with doctors / physicians that included face-to-face contact. Please do not count the current visit in the emergency department.* *You can select multiple answers. Please do also provide the number of visits*	

			How often?
Family Doctor / General Practitioner	Yes	☐	_______ times
	No	☐	
	I don't know / prefer not to say	☐	
Medical specialist in an ambulatory setting	Yes	☐	_______ times
	No	☐	
	I don't know / prefer not to say	☐	
Pulmonologist / Lung specialist	Yes	☐	_______ times
	No	☐	
	I don't know / prefer not to say	☐	
Emergency department (exclude the current visit)	Yes	☐	_______ times
	No	☐	
	I don't know / prefer not to say	☐	
Inpatient treatment in a hospital	Yes	☐	**For how long?** Total length of stay / stays? in days: _______________
	No	☐	
	I don't know / prefer not to say	☐	

15A. In case you visited an emergency department room in in the last 6 months:

Please do only answer this question if you have visited an emergency department in the past 6 months (not counting today's visit!). If not, please continue with the next question "In the past 6 months, did you use the emergency house call service…"

How many different emergency departments did you visit in the past 6 months? (not counting today's visit)	Number: __________________
What were your complaints	☐ Same or similar complaints as today

	Other complaints, namely: ________________
I don’t know / prefer not to say	☐

16. In the <u>last 6 months</u>, did you use the emergency house call service provided by the “Kassenärztliche Vereinigung” ("pink cars") - and if yes, how often?

Yes	☐
If yes, how often?	_______ times
No	☐
I don’t know / prefer not to say	☐

Please do only answer the following three questions if you have a Family Doctor / General Practitioner. If you don’t have a Family Doctor / General Practitioner, please continue with question No. 20 “Who made the decision that you come to the emergency department” on page 10.

17. For how many years have you been a patient with your Family Doctor / General Practitioner?

Please select only one answer.

One year or less	☐
More than one and up to five years	☐
More than five and up to ten years	☐
More than ten years	☐
I don’t know / prefer not to say	☐

18. How satisfied are you with the care provided by your Family Doctor / General Practitioner?

Please select only one answer.

Very satisfied	☐
Satisfied	☐
Neither satisfied not dissatisfied	☐
Dissatisfied	☐
Very dissatisifed	☐
I don’t know / prefer not to say	☐

19. The following statements are about your (own) General Practitioner:

Pseudonym (EMAxy_Zentrum_00xy): Date of interview:

Please rate each statement and select the category that matches best.						
I know my General Practitioner very well						
	☐	☐	☐	☐	☐	☐
	Strongly agree	Agree	Neutral	Disagree	Strongly disagree	I don't know / prefer not to say
My General Practitioner knows my medical history very well						
	☐	☐	☐	☐	☐	☐
	Strongly agree	Agree	Neutral	Disagree	Strongly disagree	I don't know / prefer not to say
My General Practitioner always knows very well what he / she did previously						
	☐	☐	☐	☐	☐	☐
	Strongly agree	Agree	Neutral	Disagree	Strongly disagree	I don't know / prefer not to say
My General Practitioner knows my family circumstances very well						
	☐	☐	☐	☐	☐	☐
	Strongly agree	Agree	Neutral	Disagree	Strongly disagree	I don't know / prefer not to say
My General Practitioner knows my daily activities very well						
	☐	☐	☐	☐	☐	☐
	Strongly agree	Agree	Neutral	Disagree	Strongly disagree	I don't know / prefer not to say
My General Practitioner contacts me if it is needed, I do not have to ask						
	☐	☐	☐	☐	☐	☐
	Strongly agree	Agree	Neutral	Disagree	Strongly disagree	I don't know / prefer not to say

My General Practitioner knows very well what I believe is important in my care						
	☐	☐	☐	☐	☐	☐
	Strongly agree	Agree	Neutral	Disagree	Strongly disagree	I don't know / prefer not to say
My General Practitioner keeps in contact sufficiently when I see other care providers						
	☐	☐	☐	☐	☐	☐
	Strongly agree	Agree	Neutral	Disagree	Strongly disagree	I don't know / prefer not to say

**20. Who made the decision that you come to the emergency department?**<br>*Please select only one answer.*	
I made the decision myself	☐
People who walked by / strangers	☐
Relatives, family members, friends, colleagues, supervisor at work	☐
Family Doctor / General Practitioner	☐
Other outpatient physician	☐
I was referred from another hospital	☐
Nursing staff	☐
Other: ____	☐
I don't know / prefer not to say	☐
**21. How did you arrive at the emergency department today?**<br>*Please select only one answer.*	
I came with an ambulance / fire brigade	☐
I was brought here by relatives / friends	☐
I drove here myself (with a motorized vehicle)	☐
I walked	☐

I came by public transport	☐
I came by taxi	☐
I don't know / prefer not to say	☐
**22. Did you try to make contact with a doctor's practice before your visit to the emergency department?**	
Yes, with a Family Doctor's / General Practitioner's practice	☐
Yes, with a Medical specialist's practice	☐
No	☐
I don't know / prefer not to say	☐
**23. Why did you decide to visit an emergency department with your current complaints?**<br>*You can select multiple answers.*	
Because the ambulance service / emergency physician decided to admit me	☐
Because my complaints were so severe	☐
Because the situation felt threatening to me / because I was scared	☐
Because my GP's practice was closed<br>☐ Night<br>☐ Weekend / Public holiday<br>☐ Vacation<br>☐ Out of office hours on a working day	☐
Because I could not reach my GP / Medical specialist<br>☐ GP<br>☐ Medical specialist	☐
Because I don't have time during the regular opening hours of doctor's practices (e.g. because of work)	☐
Because I could not get a timely appointment with my GP or specialist, although I tried to. You can select multiple answers.<br>☐ GP<br>☐ Medical specialist	☐
Because I didn't want to wait as long as in a doctor's practice (in the waiting area)	☐
Because I will receive better treatment compared to a doctor's practice	☐

Because the workflow is well-organized in the hospital	☐
Because diagnostic and therapeutic options are more comprehensive in the hospital (e.g. X-ray, laboratory)	☐
Because I wanted a second opinion	☐
Because I am just visiting this city	☐
Because I have moved to Berlin just recently and don't know any doctors yet	☐
Because the results of investigations are available more quickly	☐
Because there are special experts at the hospital	☐
Because the emergency department is always open and no appointment is necessary	☐
Because the ED is closer to my home than a practice	☐
Because the emergency department can be reached easier than the doctor's practice (public transport, parking lots…)	☐
Because I have visited the emergency department before and was satisfied with the treatment	☐
I don't know / prefer not to say	☐
Other reasons: ____	☐
**24. From your perspective, would a Family Doctor / General Practitioner also have been able to solve your problem?**	
Yes	☐
No	☐
I don't know / prefer not to say	☐
**25. Why did you choose to come to this specific emergency department?** *You can select multiple answers.*	
The ambulance team or referring doctor decided or recommended it	☐
It is conveniently located and easy to reach	☐
Near to my home / work	☐
This emergency department / hospital has a good reputation	☐
Medical specialists / specialized departments are available	☐

Specialized diagnostic procedures are available	☐
I was a patient here before and the staff knows me	☐
I was a patient here before and was satisfied with the treatment	☐
I don’t know / prefer not to say	☐
Other reasons:____	☐
**26. How satisfied are you with your current treatment in the emergency department?**<br>*Please select only one answer.*	
Very satisfied	☐
Satisfied	☐
Neither satisfied nor dissatisfied	☐
Dissatisfied	☐
Very dissatisfied	☐
I don’t know / prefer not to say	☐
**27. If you are dissatisfied with your current treatment in the emergency department: What are the reasons for your dissatisfaction?**<br>*You can select multiple answers.*	
My complaints were not taken seriously	☐
I should have been admitted to inpatient treatment	☐
Waiting time was too long	☐
Workflow in the emergency department was badly organized	☐
Staff was unfriendly	☐
Unneccessary procedures / examinations were performed	☐
Necessary procedures / examinations were not performed	☐
I do not believe in the accuracy of the diagnosis I received	☐
I did not receive sufficient treatment for my acute symptoms / pain	☐
I received unneccessary treatment	☐

I did not receive any (or not enough) medication for the time after discharge	☐
I did not receive enough information (concerning my treatment, next steps to take)	☐
I could not communicate enough with my relatives	☐
Communication with staff was limited due to language barriers	☐
I don't know / prefer not to say	☐
Other reasons: ____	☐
**28. What will you do after you are discharged from the emergency room?** *Please select only one answer.*	
Nothing further	☐
I will visit my Family Doctor / General Practitioner for further examinations or treatment	☐
I will visit a Medical specialist for further examinations or treatment	☐
I can't tell at the moment, because the treatment in the emergency department is not finished yet	☐
Other: ____	☐
I don't know / prefer not to say	☐
**By now, you have completed the greater part of the questionnaire. In the following section, we would like to ask you some more questions about yourself and your personal life situation.**	
**29. In which country were your parents born?**	
**My mother…**	
was born in Germany.	☐
was born in another country.	☐
If your mother was not born in Germany, which country was she born in? ____	
I don't know / prefer not to say	☐

**My <u>father</u>…**	
was born in Germany.	☐
was born in another country.	☐
If your father was not born in Germany, which country was he born in?<br><br>____	
I don’t know / prefer not to say	☐
**30. Were you born in Germany?**	
Yes	☐
No	☐
If you were not born in Germany, which country were you born in?<br><br>____	
I don’t know / prefer not to say	☐
**30 A. If you were not born in Germany: Since when do you mainly live in Germany? Please provide the year.**	
Since (YYYY): ________________________	
I don’t know / prefer not to say	☐
**31. What is your <u>highest</u> level of school education?**	
Middle school / Junior High School	☐
GCSE / 10th grade or similar	☐
A-levels / High school diploma	☐
Still attending school	☐
No school-leaving certificate / no diploma	☐
None of the above mentioned applies / other school education	☐
I don’t know / prefer not to say	☐

**31A. For how many years have you attended school?**	
Duration of school attendance (in years): ___________________________________	
I don't know / prefer not to say	☐
**32. What is your <u>highest</u> professional degree / qualification?**	
Vocational training / apprenticeship completed	☐
Academic education (university / college / doctorate)	☐
Still in vocational training / apprenticeship	☐
Still attending college or university	☐
No professional degree	☐
None of the above mentioned applies / other professional degree: ____	☐
I don't know / prefer not to say	☐
**33. Which of the following applies to your current professional situation?**	
Employed	☐
Self-employed	☐
On permanent leave (e. g. maternity leave, parental leave)	☐
Retired	☐
Unemployed	☐
Permanently unable to work	☐
Housewife / househusband	☐
Student	☐
Other: ____	☐

I don't know / prefer not to say	☐
**34. How many hours per week do you usually work? Please indicate only paid working hours.**	
Working hours per week in total (e. g. 37.5 hours): ___ , ___ hours	
I don't know / prefer not to say	☐
**35. Where do you currently live?**	
In my own apartment / flat, or house (own property, renting, or with relatives)	☐
Assisted living (e.g. retirement home, residence for seniors etc.)	☐
Nursing home / special care home	☐
Without permament residence / homeless	☐
Accommodation for refugees	☐
Other: ____________________________________	☐
I don't know / prefer not to say	☐
**36. How many people live in your household?** Please count yourself, spouse, children, and room mates	
1 person, i.e. only yourself	☐
Total of ________ persons	
I don't know / prefer not to say	☐
**37. How many people are so close to you that you can count on them if you have serious problems?**	
None	☐
1 or 2	☐
3 to 5	☐
More than 5	☐
I don't know / prefer not to say	☐

**38. What is your marital status? Which of the following applies?**	
Married, living together with spouse	☐
Married, living permantly separated	☐
Single (never married before)	☐
Divorced	☐
Widowed	☐
I don't know / prefer not to say	☐
**39. Do you have a life partner?**	
Yes	☐
No	☐
I don't know / prefer not to say	☐
**Now we would like to ask you some questions concerning your health and care situation.**	
**40. Do you receive long-term care?**	
Yes	☐
No	☐
I don't know / prefer not to say	☐
**40A. If yes, do you receive long-term care in the German health care system according to the system of „Pflegestufe" or „Pflegegrad"? If yes, please indicate below:**	
1a Pflege**stufe** (1-3): ___ ___	
1b Pflege**grad** (1-5): ___ ___	
I don't know / prefer not to say	☐
**41. Do you currently smoke?**	
Yes	☐

No	☐
Ex-Smoker	☐
I don’t know / prefer not to say	☐

**41A. What is your body weight?**	______ kg
**41B. What is your body height?**	______ cm
I don’t know / prefer not to say	☐

42. Now we would like to know how good of bad your health is TODAY.

This scale is numbered from 0 to 100.

100 means the best health you can imagine, 0 means the worst health you can imagine.

Mark an X on the scale to indicate how your health is TODAY.

Now, please additionally write down the number you marked on the scale here.

YOUR HEALTH TODAY = __________

Best health you can imagine.

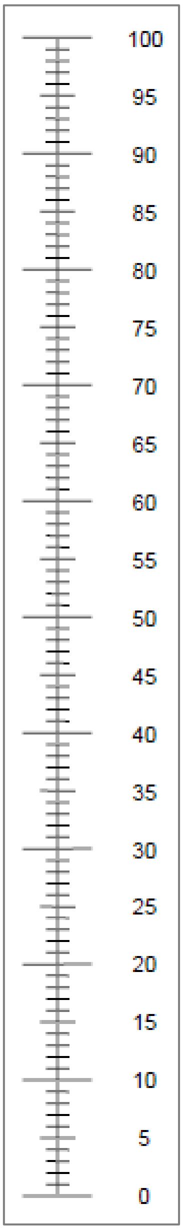

Worst health you can imagine.

**43. Finally, we would like to know how satisfied you currently are with your life.**<br><br>**All things considered, how satisfied are you with your life these days? Please indicate on the scale below from 0 to 10.**	
Not satisfied at all. Very satisfied.<br><br>o---------o---------o--------o---------o---------o---------o---------o--------o---------o---------o<br>0 1 2 3 4 5 6 7 8 9 10	
I don’t know / prefer not to say	☐
